# The Nucleic Acid Knowledgebase: a new portal for 3D structural information about nucleic acids

**DOI:** 10.1093/nar/gkad957

**Published:** 2023-11-11

**Authors:** Catherine L Lawson, Helen M Berman, Li Chen, Brinda Vallat, Craig L Zirbel

**Affiliations:** Institute for Quantitative Biomedicine, Rutgers, State University of New Jersey, 174 Frelinghuysen Road, Piscataway, NJ 08854, USA; Research Collaboratory for Structural Bioinformatics Protein Data Bank, Rutgers, The State University of New Jersey, Piscataway, NJ 08854, USA; Research Collaboratory for Structural Bioinformatics Protein Data Bank, Rutgers, The State University of New Jersey, Piscataway, NJ 08854, USA; Department of Chemistry and Chemical Biology, Rutgers, The State University of New Jersey, Piscataway, NJ 08854, USA; Department of Quantitative and Computational Biology, University of Southern California, Los Angeles, CA 90089, USA; Institute for Quantitative Biomedicine, Rutgers, State University of New Jersey, 174 Frelinghuysen Road, Piscataway, NJ 08854, USA; Research Collaboratory for Structural Bioinformatics Protein Data Bank, Rutgers, The State University of New Jersey, Piscataway, NJ 08854, USA; Institute for Quantitative Biomedicine, Rutgers, State University of New Jersey, 174 Frelinghuysen Road, Piscataway, NJ 08854, USA; Research Collaboratory for Structural Bioinformatics Protein Data Bank, Rutgers, The State University of New Jersey, Piscataway, NJ 08854, USA; Department of Mathematics and Statistics, Bowling Green State University, Bowling Green, OH 43403, USA

## Abstract

The Nucleic Acid Knowledgebase (nakb.org) is a new data resource, updated weekly, for experimentally determined 3D structures containing DNA and/or RNA nucleic acid polymers and their biological assemblies. NAKB indexes nucleic acid-containing structures derived from all major structure determination methods (X-ray, NMR and EM), including all held by the Protein Data Bank (PDB). As the planned successor to the Nucleic Acid Database (NDB), NAKB’s design preserves all functionality of the NDB and provides novel nucleic acid-centric content, including structural and functional annotations, as well as annotations from and links to external resources. A variety of custom interactive tools have been developed to enable rapid exploration and drill-down of NAKB’s content.

## Graphical Abstract

**Graphical Abstract:**
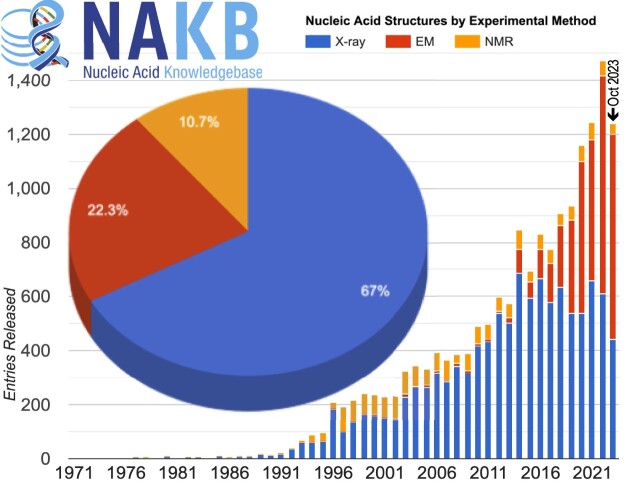

## Introduction

The first three-dimensional (3D) nucleic acid (NA) structures were determined in the 1970s and 1980s, with early investigations focused on small DNA fragments and transfer RNA (1). A half-century later, the number, diversity and complexity of NA-containing 3D structures in the Protein Data Bank (PDB) (2,3) has grown substantially and continues to do so at an accelerating rate. More than 16000 structures are now available, generated by investigators from around the world using X-ray crystallography (X-ray), nuclear magnetic resonance (NMR) or cryo electron microscopy (EM) methods. Facilitated in large part by advancements in EM, many large complexes are now available that feature combinations of polymerases, ribosomes, nucleosomes, and/or spliceosomes, providing unprecedented insights into fundamental biological processes (4).

Nucleic acids have diverse functional roles, from storing genetic information and regulating expression to sensing cellular changes and catalyzing reactions. DNA encodes genetic information and takes part in replication, recombination, transcription and repair. RNA transmits genetic information, takes part in all aspects of post-transcriptional regulation, and is the key component of the ribosome, the universal protein-making nano-machine (5). Large numbers of non-coding RNAs (ncRNAs), many of them highly structured, have been discovered and many are involved in gene regulation (6). The widely recognized CRISPR-Cas systems create RNA/DNA hybrid structures to enable precision gene editing, one of many examples of biologically active nucleic acids (7).

Nucleic acids also have substantial structural variability. While DNA typically pairs with complementary strands to form long helices, more complex motifs have also been observed (8,9). RNA folds on itself to form short helices punctuated by structural motifs, e.g. hairpin, internal, and multi-helix junction ‘loops’, that stabilize complex 3D architectures and mediate binding of proteins, small molecules and other RNAs (10,11).

The Nucleic Acid Database (NDB), the first comprehensive data resource for 3D structures of nucleic acids, was developed in the 1990s at Rutgers University to support ongoing collaborative studies that used both experimental and computational approaches (1,12,13). Operating as a value-added database for more than three decades, NDB collected and maintained information about nucleic acid structures derived from X-ray and NMR in a SQL-relational database. In addition to structures and data collected from the PDB and from the Cambridge Structural Database (CSD) (14), NDB provided its own deep curation and data visualizations.

Beginning in 2019, we initiated development of the Nucleic Acid Knowledgebase (NAKB), a modern successor to NDB, with the following goals: (i) preserve and substantially build upon NDB’s functionality, (ii) include structures produced using EM, (iii) create a more complete and more consistent set of functional and structural annotations and (iv) link to a wider range of nucleic acid-centered external resources. A beta version of NAKB was announced in July 2022 and the fully functional service was officially launched in May 2023. NDB was retired in July 2023.

The NAKB website (nakb.org) offers a variety of search tools to quickly identify structures of interest. Also offered are tabular reports of search results, 2D fold and 3D structure visualization, education and standards pages, and an updated nucleic-acid community web + software resource list. NAKB’s website is built using modern web architecture elements for an enhanced user experience, enabling faster retrieval of search results and use on both large and small devices. The website is updated weekly on Thursdays at noon, US Eastern.

## NAKB content and tools

NAKB currently indexes 16000+ nucleic acid-containing (NA) structures, including all experimentally determined NA structures available in PDB, and 48 early structures, mostly DNA and RNA dinucleotides that were originally deposited into CSD in the 1970s through 1990s and are not available in PDB.

### Statistics

**Figure 1.**
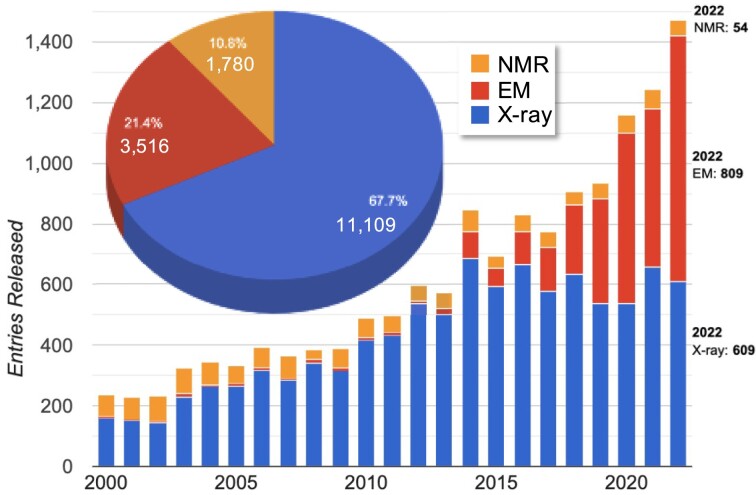
NAKB statistics: structure determination method. Pie Chart Inset: Total cumulative entry counts and percentages for each method (as of July 2023). Graph: trend for entries released between the years 2000 and 2022. Image source: NAKB Custom Charts Tool.

The Custom Charts Tool provides basic statistics on current indexed structures. Users can view entry counts reported by structure determination method, by polymer composition, or by NA composition, provided as annual trend and summary pie charts. By method (Figure 1), X-ray comprises 2/3 of all structures, with EM and NMR comprising 21% and 11%, respectively. Notably, EM use has led to a substantial increase in the number of NA structures released per year; in 2022 EM surpassed X-ray in annual counts.

**Figure 2.**
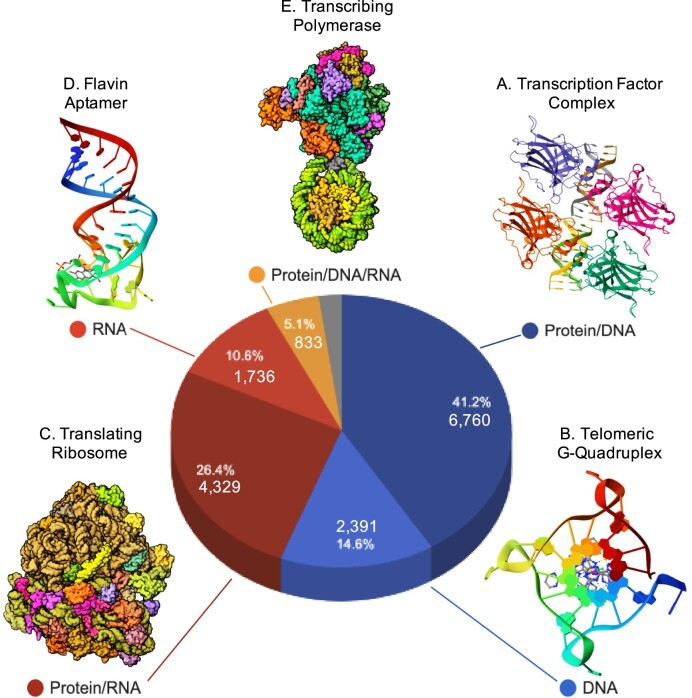
NAKB statistics: polymer composition. Pie chart indicates entry counts and percentages for the five most prevalent polymer compositions. (A–E) Recently released examples for each type. (**A**) Protein/DNA (X-ray): p73 transcription factor DNA-binding domains bound to DNA, PDB id 7EZJ (15). (**B**) DNA (X-ray): Telomeric DNA G-Quadruplex, 7QVQ (16). (**C**) Protein/RNA (EM): translating bacterial ribosome at 1.55 Å, 8B0X (17). (**D**) RNA (NMR): Flavin Aptamer, 7RWR (18). (**E**) Protein/DNA/RNA (EM): RNA polymerase II transcribing a chromatosome, 8H0V (19). Pie chart: NAKB Custom Charts. Structure images: RCSB PDB.

NAKB provides its own high-level classification for each structure entry according to the polymer composition *of the studied sample*. The classification is assigned manually when 3D coordinates are incomplete; for instance, early ribosome structures with only RNA coordinates are nonetheless classified as Protein/RNA. The most prevalent polymer compositions (Figure 2) are Protein/DNA (41%), Protein/RNA (26%), DNA (15%), RNA (11%) and Protein/DNA/RNA (5%). Minor polymer compositions (2%, Figure 2, grey wedge) include Hybrid (DNA and RNA nucleotides in the same polymer), PNA (peptide backbone NA) and Other (other backbone) NA types.

### NA and protein annotation

The structures shown in Figure 2 illustrate the wide diversity of NAKB’s content in terms of biological function: transcription factors, telomeric G-quadruplex DNA, translating ribosomes, RNA aptamers, and transcribing polymerase complexes. To ensure that users can quickly find relevant entries either by a specific biological function, or by an NA structure feature (e.g. antiparallel B-form helix, triple helix, or Holliday junction), we have completely refactored and have partially automated the mostly manual curation system that was previously used by NDB for X-ray and NMR structures (13). Annotations have also been extended to include all EM structures. On a weekly basis, NAKB assigns annotations to the NA and protein polymer entities of all newly released PDB entries. After calculating protein and NA sequence clusters using CD-HIT and CD-HIT-EST programs (20), respectively, polymer entities that fall into sequence clusters with consensus functional annotations are automatically assigned. Similarly, after calculating NA backbone, base-pair, and helix parameters for each biological assembly using DSSR (21), NA polymers with parameters that meet conservative pre-defined criteria for structural annotations are automatically assigned. Automated annotations are manually reviewed by NAKB team members who have expertise in structural biology of nucleic acids and NA complexes; missing annotations are manually assigned prior to public release.

**Figure 3.**
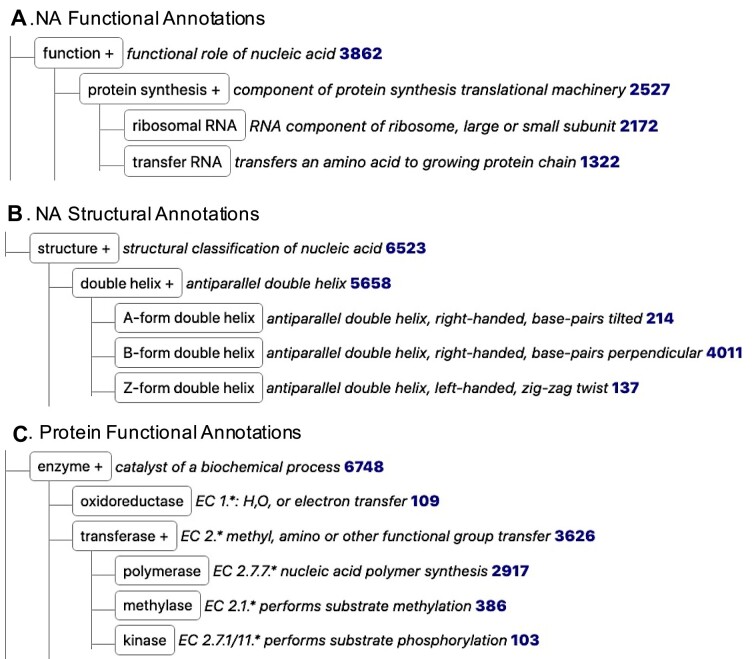
NAKB Annotation Trees Tool: selected branches. (**A**) NA function protein synthesis branch. (**B**) NA structure double helix branch. (**C**) Protein function enzyme transferase branch. Each term appears in its place within a collapsible, expandable, searchable annotation hierarchy. A short definition and structure entry count with hyperlink to the corresponding entries list is also provided.

The NAKB Annotation Trees Tool provides a comprehensive web interface for browsing and interrogating NAKB-assigned NA and protein annotations (Figure 3). Two hierarchical dictionaries have been built to support the curation system and Trees Tool. The NA annotation dictionary contains 55 defined terms (39 functional, 16 structural) within these major categories:

- functional: protein synthesis, catalytic, riboswitch, aptamer, translation regulating, transcription regulating, post-translational processing, replication regulating, telomeric DNA
- structural: double helix, parallel helix, triple helix, quadruplex, Holliday junction, feature (e.g. cyclic), designed assembly

The protein annotation dictionary contains 71 defined terms organized in enzyme, regulatory and structural categories:

- enzyme: oxidoreductase, transferase, hydrolase, lyase, isomerase, ligase, recombinase
- regulatory: transcription, CRISPR-Cas, cell cycle, cell signaling, DNA replication/repair, gene silencing, immune system, post-transcriptional, toxin/antitoxin, translation
- structural: chromatin, ribonucleoprotein, virus

The Annotation Trees Tool has custom features that provide the ability to investigate NA annotations by NA class (e.g. DNA-only, RNA-only), and to show the most highly populated protein/regulatory branch, which is transcription factors. NA-associated PANTHER Protein Class annotations (22) are also available in a separate tab of the tool (not shown). Each structure count links to a Search page listing all structures with the indicated annotation.

### Other annotations

The NAKB Annotation Tables Tool enables users to view, sort, search and download distributions of data values for >20 data items with textual or database identifier values that are collected and aggregated by NAKB. Data sources are from NAKB, PDB or other external resources, as shown in Table 1. For each data value, the tool provides the current structure entry count with hyperlink to the corresponding search result list. For example, selecting ‘Nonstandard NA Residues’ yields a table with > 800 nonstandard NA residues, listed as PDB CCD ids (23). Corresponding entry counts link to lists of entries containing the nonstandard residue (e.g. entries containing pseudouridine (PSU): nakb.org/?nakblist=nonstandard:PSU).

**Table 1.** Data items for which searchable/sortable/downloadable data distributions can be obtained via the NAKB Annotation Tables Tool

Type	Data item	Example value	Source^a^	Ref.
Polymer	Composition	Protein/DNA/RNA	NAKB	
	Type	Protein	NAKB	
Nucleic Acid	Annotation	Double helix	NAKB	
	Composition	DNA + RNA	NAKB	
	Nonstandard NA Residues	PSU	PDB	(23)
RNA	Equivalence Class (RNAEQ)	NR_all_83717.120	bgsuRNA	(24)
	RNAcentral id/name	URS0000049E57_562/rRNA	RNAcentral	(25)
	Rfam id/name	RF00001/5S ribosomal RNA	Rfam	(26)
Protein	Annotation	Transferase	NAKB	
	PANTHER protein class	DNA metabolism protein/PC00009	PANTHER	(27)
	UniProt id	P60438	PDB	
Ligand	Nonpolymer id/name	ATP/ADENOSINE-5′-TRIPHOSPHATE	PDB	(23)
Experiment	Method (abbr)	X-ray	PDB	
	Method (full)	X-RAY DIFFRACTION	PDB	
	Crystal Space Group	*P* 21 21 2	PDB	
	Software	PHENIX	PDB	
Entry	Author	Steitz TA	PDB	
	Deposition Site	RCSB	PDB	
Citation	Journal	Nucleic Acids Res	PDB	
	PubMed id	35662248	PDB	
	DOI	10.1126/science.add9633	PDB	

^a^Indicates data aggregation source used in NAKB’s pipeline. All PDB metadata is obtained via the RCSB PDB API service (28).

### Search

**Figure 4.**
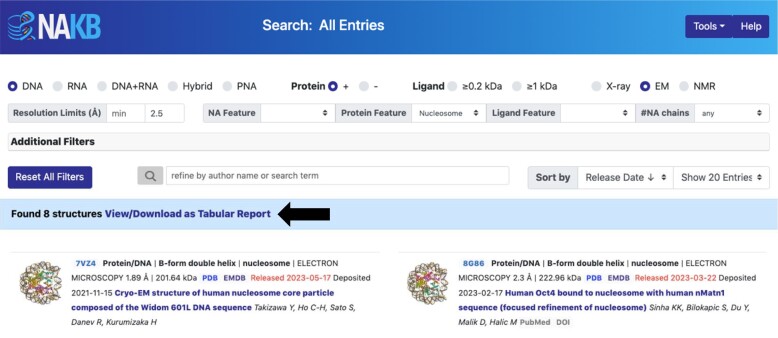
NAKB Search example. The results banner shows that 8 structures meet selected criteria: NA type = DNA, has protein, method = EM, resolution ≤2.5 Å, and protein feature = nucleosome. The ‘View/Download as Tabular Report’ link (black arrow) enables generation of custom reports for the current result set.

NAKB’s Search tool enables rapid exploration and drill-down of the entire archive (Figure 4). It combines and expands upon functionality previously split across three separate search tools (called DNA, RNA, Advanced) in NDB. Basic search options are displayed at top, e.g. NA composition, with or without protein, ligand present by molecular weight (MW) cutoff, experimental method, resolution limits. Advanced options can be accessed by toggling ‘Additional Filters’. Each filter selection interactively updates the results list; all selections can be quickly set back to default values to facilitate testing of alternative strategies. Results can be sorted by resolution, deposited MW, release date, id, or dinucleotide conformation (DNATCO) score (29). Sorting by deposited MW is particularly useful for navigating results lists that contain both large multi-component complexes (e.g. translating ribosomes) and smaller individual components (e.g. transfer RNAs). Alternatively, one can apply additional filtering options to limit results to the desired complexity state (e.g. Protein +/–, #NA chains, deposited MW min/max).

The URL for NAKB Search is nakb.org/nakblist=all. Optionally, ‘all’ can be replaced with another term recognized by the search engine (Apache Solr) to produce a subset of structures for further filtering. This feature is heavily utilized by other NAKB tools to generate custom result lists, including the website ‘Quick Search’ box, home page Recent Entries carousel (nakb.org/nakblist=latest), Custom Charts, Annotation Trees, Annotation Tables and Individual Entry Atlas pages (described below).

### Tabular reports

A variety of tabular reports can be created and downloaded for any search result set by clicking on ‘View/Download as Tabular Report’ in the Search results banner (Figure 4, black arrow). Status/Citation, Method, and Polymer Components report types can be customized to add or remove data columns of interest from a large set of options, and can be downloaded in excel, csv, and json formats. Backbone Torsions, Sugar Torsions, Base Pairs and Base Pair Steps report types contain values pre-calculated using DSSR (21), and can be downloaded in CSV format.

### Individual entry atlas pages

**Figure 5.**
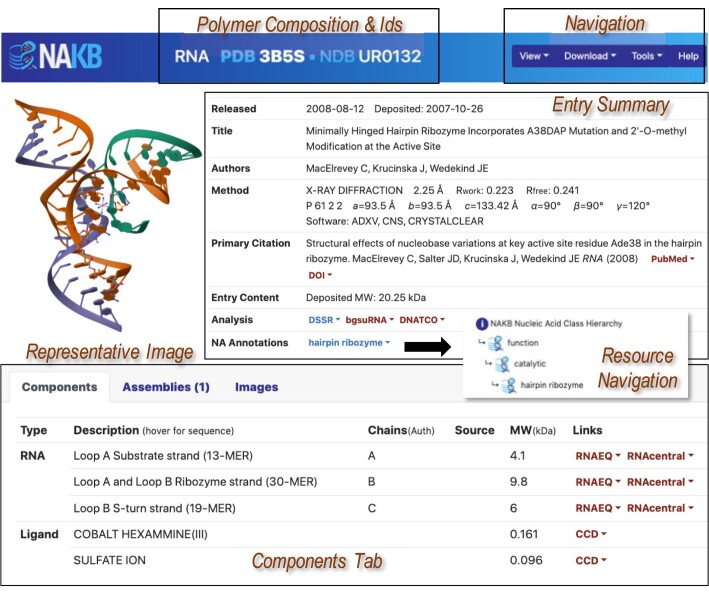
NAKB Atlas page, annotated to highlight key sections.

Dynamically generated individual Entry Atlas pages (Figure 5) display aggregated content for each structure entry according to its assigned ids (URL nakb.org/atlas=*id*, either PDB id or NDB id can be entered). Each Atlas page includes: (i) a Header that states the entry's polymer composition and assigned id(s); (ii) Navigation options to view structure using Mol* (30), download coordinates and PDB validation report from RCSB PDB if PDB entry, otherwise download coordinates from NAKB, and access other tools or NAKB help; (iii) a Summary Table that lists entry-level data items, annotations and links to internal and external analysis options; (iv) a Components Tab that lists and provides summary information for all unique polymers and non-polymer components (entities); (v) an Assemblies Tab (not shown) that describes each defined biological assembly of the deposited structure in terms of chain selections, number of symmetry operations, symmetry type, with action links to view the assembly in 3D with Mol* (30), access precalculated DSSR NA-parameters (URL nakb.org/naparams.html?*id_#*, where # is the assembly number) (21), or download assembly coordinates; (vi) an Images Tab (not shown) that provides additional images, including base-pairing diagrams produced by RNAView (31).

Atlas pages provide embedded resource navigation menus in multiple locations, colored either blue (for internal NAKB service) or red (for external resource). All menus open on hover to provide a short resource description and one or more navigation links. In Figure 5, ‘hairpin ribozyme’ links to the search result list for all entries with that functional annotation. In addition to NAKB NA and protein annotations, resource navigation menus provide information about/access to the following resources:

- experimental data: BMRB (32), EMDB (33)
- primary citation: PubMed, DOI
- analysis/internal: DSSR NA parameters (21), R2DT diagrams (34)
- analysis/external: bgsuRNA (10), DNATCO (35), DNAproDB (36), 3DFootPrint (37), ONQUADRO (38), G4DSSR (21),
- RNA components: RNAEQ (24), RNAcentral (25), Rfam (26)
- Protein components: UniProt (39)
- Ligand components: PDB CCD (23), PDB BIRD (40)

### External resources list

NAKB maintains a searchable/sortable table with more than 50 external resources for 3D structural analyses of NA and their complexes. Each resource is classified according to the polymer type(s) analyzed (DNA, RNA, protein) and service type (e.g. web server, analysis of PDB structures, data archive, software package, community activity). The table merges NDB’s Tools and Software lists. Suggestions for additions and corrections by the scientific community are welcomed.

### Education

NAKB offers several education pages that expand upon those offered by NDB. Introduction to Nucleic Acids provides basic introductory information about DNA, RNA, basepairs, and A/B/Z double helical forms. The RNA Basepair Catalog provides interactive examples of each basepair class according to the Leontis-Westhof classification system (41). Additional pages list and link to NA-related Molecule of the Month articles from RCSB PDB (42), and cover images with educational captions that NDB/NAKB has created for the journal *RNA* over the past few years. The Musical Atlas page provides unique ways to explore DNA structures: basepairing, conformation and other parameters are converted into discrete notes, rhythms, and pitches to yield simple melodies.

### Standards

NAKB’s Standards menu has three pages. Valence Geometry lists bond length and bond angle standard values derived from high resolution structure surveys (43,44). The Nucleotides page provides standard IUPAC nomenclature definitions for sugar-phosphate backbone torsion angles, sugar torsion angles and pucker, N-glycosidic bond anti vs. syn, as well as definitions for virtual torsion angles (45) and dinucleotide conformer classes (29). The Base Pairs page describes Leontis-Westhof and Saenger basepair classifications, and provides illustrations for each of the basepair and basepair step parameters that are commonly used in NA conformational analysis (46).

## Data pipeline

**Figure 6.**
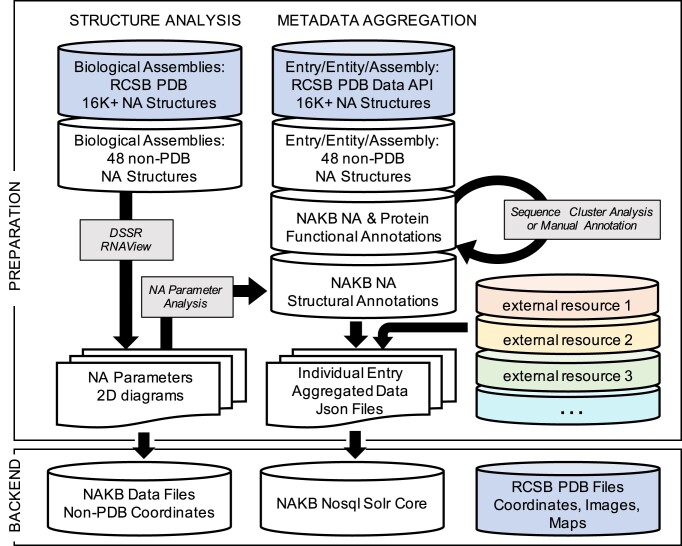
Schematic overview of NAKB’s system to prepare nucleic acid 3D structure data for web delivery. Data resources (disks) with external dependencies are shaded (see Table 2 for external resource list). Black arrows & grey boxes represent scripted processes that prepare individual files for loading onto website backend services.

NAKB’s backend infrastructure consists of a data preparation pipeline that loads structural analysis results to web-accessible file storage and aggregated metadata to an Apache Solr core (Figure 6). Solr is a NoSQL-style open-source search engine written in Java that provides full-text and faceted search, real-time indexing and dynamic clustering.

In designing the new resource, we prioritized preservation and improvement of unique services offered by NDB while avoiding unnecessary duplication. Rather than prepare and maintain a full independent system of atomic coordinate files in multiple formats (PDB and mmCIF) and images of all indexed structures, as was done by NDB, NAKB maintains coordinates, metadata and images only for the 48 crystal structures previously indexed by NDB that are not available in PDB. For all PDB structures, NAKB links to coordinate, image, and experimental density map files maintained by our Research Collaboratory for Structural Bioinformatics partner, RCSB PDB (47) and obtains all PDB-derived metadata and current UniProt accession ids (39) via RCSB PDB’s Data API (28) (Figure 6, blue shading).

External NA-centric resources were identified through NAR’s Molecular Biology Database Collection, NDB’s resource list, and literature search. We prioritized active, regularly updated resources focusing on one or more aspects of sequence, structure, function and/or interactions of nucleic acids found in 3D structures and their complexes. Current external resources are listed in Table 2. NAKB interconnects with each of these resources at either the structure entry or structure component level by providing direct links from entry atlas pages to the external resource's website.

**Table 2.** External metadata resources for the NAKB pipeline

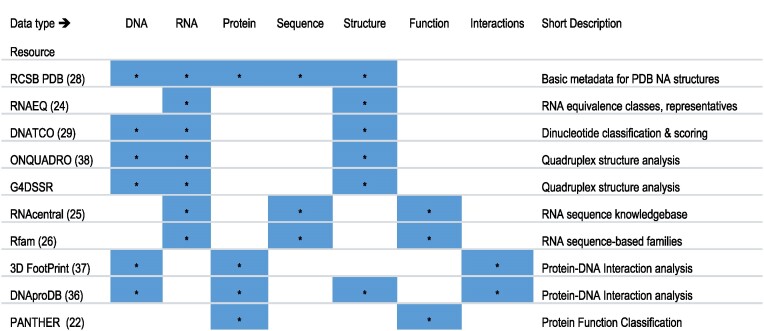

### Data preparation

NAKB’s preparation system has two tracks, Structure Analysis and Metadata Aggregation. All processes are controlled by custom bash shell and Python 3 scripts.

In the Structure Analysis track, biological assemblies belonging to each indexed structure are systematically analyzed using the programs DSSR (21) and RNAView (31). For PDB entries, mmCIF-format biological assembly files that are directly available from PDB are used. Nucleic acid parameters collected from DSSR output include nucleotide backbone and sugar ring torsion angles, Leontis-Westhof, Saenger and DSSR base-pair classifications, as well as base-pair and base-pair step geometries (21). Parameters are collected into CSV-format files for web distribution. RNAView 2D folding diagrams are generated for smaller structures. RNAView's native postscript output is converted to SVG format for web display using Inkscape.

In the Metadata Aggregation track, a json formatted file is generated for each indexed structure that contains relevant metadata collected from all data resources (RCSB PDB Data API for PDB entries, NAKB data files for non-PDB entries, plus external resources as listed in Table 2). The aggregated data are loaded into an Apache Solr core to enable web query.

Each external data resource file is first imported in its native format. For ease of data integration, the native format is in most instances converted to an intermediate json file with data items indexed by data-type appropriate key, either entry id, or entry + polymer entity id. For resources that natively assign data by chain id, such as RNAEQ, Rfam, and RNAcentral, NAKB internally reindexes the data to the corresponding polymer entity id.

### Implementation

NAKB’s web service is installed on mirror US East and West coast virtual Ubuntu Linux + Apache 2 servers that reside within RCSB PDB’s cyberinfrastructure system at Rutgers University and UC San Diego (47).

## Discussion

The Nucleic Acid Knowledgebase is a new, custom designed web resource for interrogating NA-containing 3D structures, featuring multiple tools that enable users to quickly find and visualize structures of interest and to investigate interrelationships of sequence, structure, function, and interactions. The data preparation pipeline streamlines aggregation of rich functional and structural annotations produced by NAKB’s curation system, key metadata from PDB, and data from multiple external NA-centered resources to support website search. The new system has been designed to readily support the anticipated continued growth and increasing complexity of experimentally determined NA structures.

## Data Availability

Data available for download from NAKB (nakb.org/download.html) include the NAKB accession id list (updated weekly), NAKB-assigned protein and NA functional and structural annotations (updated weekly), legacy NDB annotations, and coordinate files for 48 early structure entries not available in PDB.
